# Predicting 10-Year Diabetes Risk Through Physiological Acceleration: A Longitudinal Deep Learning Ensemble Approach

**DOI:** 10.3390/diagnostics16070992

**Published:** 2026-03-25

**Authors:** Sangsoo Kim, Seonghee Park, Jinmi Kim, Ha Jin Park, Soree Ryang, Myungsoo Im, Doohwa Kim, Kyeongjun Lee

**Affiliations:** 1 Division of Endocrinology and Metabolism, Department of Internal Medicine, Pusan National University Hospital, Busan 49241, Gyeongsangnam-do, Republic of Korea; 2 Biomedical Research Institute, Pusan National University Hospital, Busan 49241, Gyeongsangnam-do, Republic of Korea; 3 Department of Internal Medicine, Pusan National University School of Medicine, Busan 49241, Gyeongsangnam-do, Republic of Korea; 4 Department of Mathematics and Big Data Science, Kumoh National Institute of Technology, Gumi 39177, Gyeongsangbuk-do, Republic of Korea; 5 Department of Biostatistics, Clinical Trial Center, Biomedical Research Institute, Pusan National University Hospital, Busan 49241, Gyeongsangnam-do, Republic of Korea

**Keywords:** Type 2 Diabetes Mellitus, deep learning, longitudinal trajectory, ensemble learning, precision medicine

## Abstract

**Background/Objectives**: Type 2 diabetes (T2D) develops gradually over many years through a prolonged preclinical phase, yet traditional static risk scores often fail to capture these dynamic metabolic trajectories. We propose a longitudinal deep learning framework to predict the 10-year risk of Type 2 diabetes onset defined by comprehensive ADA criteria by modeling the physiological acceleration of routine clinical biomarkers. **Methods**: Utilizing an 18-year longitudinal dataset from the community-based Korean Genome and Epidemiology Study (KoGES) cohort, we selected N=4354 participants with complete follow-up records, ensuring high data integrity without requiring synthetic data augmentation. We constructed a 3-dimensional tensor of 21 non-invasive clinical variables spanning a 6-year observation window. To resolve the inherent precision-recall trade-offs of individual models, we developed a stacking ensemble that integrates Long Short-Term Memory (LSTM) and Gated Recurrent Unit (GRU) architectures via a logistic regression meta-learner. To evaluate the added value of longitudinal modeling, we compared this dynamic framework against a static XGBoost baseline that only saw the most recent data. **Results**: Evaluated on an independent test set (n=874), the ensemble significantly outperformed baseline models, achieving an overall accuracy of 0.90 (95% CI: 0.88–0.92) and an AUROC of 0.94 (95% CI: 0.93–0.95). By harmonizing LSTM’s sensitivity and GRU’s precision, the model yielded an exceptional Positive Predictive Value (PPV) of 0.97, a sensitivity of 0.80, and a specificity of 0.98. **Conclusions**: This framework provides a highly accurate, resource-efficient triage instrument for T2D screening, thereby reducing unnecessary clinical alerts and improving screening efficiency.

## 1. Introduction

### 1.1. Global Burden and the Need for Early Stratification

Diabetes Mellitus (DM) has evolved into a critical global epidemic, imposing a substantial burden on healthcare systems and individual quality of life. According to the International Diabetes Federation (IDF), the global prevalence of diabetes is projected to reach 783 million by 2045 [1]. The insidious nature of Type 2 Diabetes (T2D), which often develops asymptomatically over several years, underscores the urgent need for early risk stratification. Identifying high-risk individuals before the onset of irreversible metabolic dysregulation enables timely interventions, such as lifestyle modifications, which are proven to delay or prevent disease progression [2].

### 1.2. Limitations of Conventional Longitudinal Analysis

In recent years, machine learning (ML) algorithms have demonstrated superior performance over conventional statistical models (e.g., Cox proportional hazards) in predicting diabetes risk [3,4]. However, a significant limitation of existing studies—even those utilizing high-quality longitudinal cohorts like the Korean Genome and Epidemiology Study (KoGES)—is their tendency to aggregate temporal data into static summary statistics (e.g., mean, slope) or to rely on a single snapshot of health status [5]. Such approaches inadvertently discard the temporal dynamics and subtle fluctuations of biomarkers that occur during the pre-diabetic phase. Since the trajectory of glucose metabolism is non-linear and highly variable among individuals, capturing the full sequential pattern of health checkup data is essential for precise prediction [6].

### 1.3. Deep Learning Approaches and the Necessity of Ensembling

To address these limitations, recurrent neural architectures, specifically Long Short-Term Memory (LSTM) and Gated Recurrent Units (GRU), have been increasingly adopted for time-series analysis in healthcare [7]. These gated architectures are specifically designed to mitigate the vanishing gradient problem inherent in standard Recurrent Neural Networks (RNNs), allowing the model to learn long-term dependencies effectively. While LSTMs are theoretically optimized for retaining historical context over extended periods, GRUs often exhibit faster convergence and greater sensitivity to immediate temporal shifts [8,9]. However, relying on a single deep learning architecture can lead to high variance in predictions and increased susceptibility to overfitting, especially given the inherent noise and heterogeneity of longitudinal physiological data [10]. Therefore, a unified framework that synthesizes diverse temporal representations is required to achieve robust generalization.

### 1.4. Study Contribution: A Robust Stacking Ensemble Framework

In this study, we propose a Stacking Ensemble Framework that strategically integrates the complementary strengths of LSTM and GRU to predict the 10-year risk of diabetes onset. We prioritized data quality over sample size. Using the KoGES cohort, we selected only participants who attended every scheduled visit, ensuring the model learned from continuous, uninterrupted disease progressions. This strict filtering naturally yielded a balanced class distribution (1899 Non-diabetes vs. 1581 Diabetes), eliminating the need for synthetic augmentation techniques like SMOTE which can introduce artifacts.

To benchmark the proposed longitudinal framework against a conventional machine learning approach, we included XGBoost as a static baseline using cross-sectional data. By leveraging the full 10-year history of high-quality, complete cases, this study offers a precise, data-driven methodology for identifying at-risk populations in community healthcare settings.

## 2. Methods

### 2.1. Study Design and Data Source

This study utilized data from the Ansan–Ansung cohort of the Korean Genome and Epidemiology Study (KoGES), a community-based prospective longitudinal study initiated by the Korea National Institute of Health (KNIH) [11]. The cohort initially recruited 10,030 participants aged 40–69 years from the urban area of Ansan and the rural area of Ansung, Republic of Korea, between 2001 and 2002 (Baseline, $T_{0}$). Follow-up examinations were conducted biennially. For this study, we analyzed longitudinal data collected over approximately 18 years, corresponding to a maximum follow-up duration of 16 years per participant ($T_{0}$–$T_{8}$, 2001–2018). The Ansan–Ansung study protocol was approved by the Institutional Review Board of the Korea Centers for Disease Control and Prevention, and all patients provided written informed consent. The study protocol was approved by the Institutional Review Board of Pusan National University (South Korea) (IRB No. 2101–007–099).

### 2.2. Experimental Design: Observation and Prediction Windows

To predict the long-term onset of Type 2 Diabetes (T2D) based on historical health trajectories, we structured the longitudinal timeline into two distinct windows:

- Observation Window (Input, *X*): Clinical data collected from the first four biennial examinations ($T_{0}$∼$T_{3}$; years 0–6, 2001–2008) were utilized as independent variables. This window allows the deep learning model to learn the dynamic temporal patterns of physiological indicators.
- Prediction Window (Target, *Y*): The incidence of diabetes was monitored during the subsequent five examinations ($T_{4}$∼$T_{8}$; years 8–16, 2009–2018). Any diagnosis occurring within this period was considered a positive outcome.

### 2.3. Study Population and Dataset Construction

Of the initial 10,030 participants, we implemented a rigorous data quality control protocol to prioritize the integrity of longitudinal trajectories. We applied multi-stage exclusion criteria as follows:

1. Exclusion of Pre-existing Diabetes: Participants diagnosed with diabetes at baseline ($T_{0}$) or during the observation window ($T_{0}$∼$T_{3}$) were excluded to strictly focus on the prediction of new-onset cases.
2. Requirement for Longitudinal Fidelity: To ensure that the model learns from continuous disease progressions, we selected only those participants who successfully attended all nine biennial examinations ($T_{0}$∼$T_{8}$).
3. Exclusion of Excessive Missing Data: Individuals with significant unrecoverable missing data in key variables were excluded.

**Figure 1:**
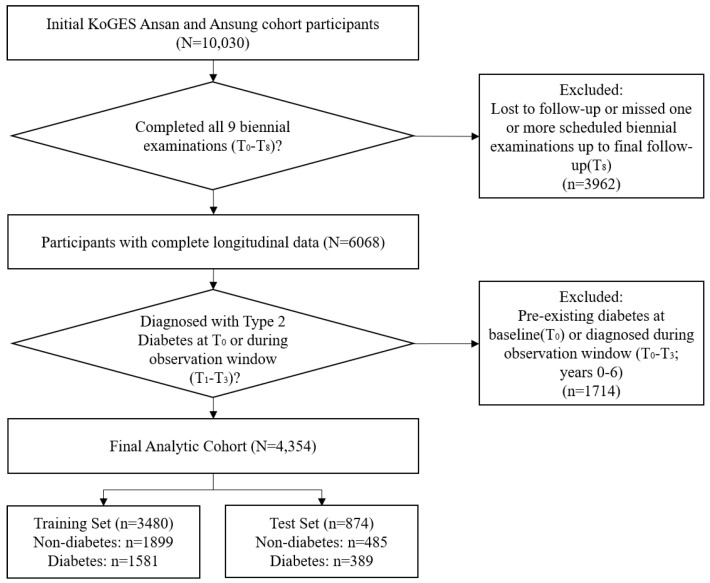
Study population selection flowchart. Note: Of the initial 10,030 participants from the KoGES Ansan–Ansung cohort, individuals with pre-existing diabetes, incomplete follow-up records, or excessive missing data were sequentially excluded. The final analytic cohort of 4354 participants was stratified into an independent training and test set.

This strict selection yielded a final analytic cohort of 4354 participants (Figure 1). The dataset was randomly split into a training set (n=3480) and an independent test set (n=874) using stratified sampling. This selection naturally yielded a well-balanced class distribution (Prevalence ≈45.2%), eliminating the need for synthetic oversampling techniques like SMOTE.

### 2.4. Clinical Feature Selection and Outcome Definition

#### 2.4.1. Input Variables: Longitudinal Feature Vector (*X*)

To model the physiological trajectory preceding the clinical onset of diabetes, we constructed a longitudinal feature vector using 21 clinical variables collected at each biennial examination during the observation window ($T_{0}$∼$T_{3}$; years 0–6, 2001–2008). These variables were carefully selected based on their established pathophysiological relevance to metabolic syndrome and insulin resistance, and were categorized into the following domains. Importantly, while 21 variables may appear extensive, they exclusively comprise the standard, universally accessible, and low-cost components of routine health screenings. This ensures that the framework can be deployed seamlessly even in resource-limited clinical settings without requiring expensive or novel biomarker assays.

- Demographic and Anthropometric Parameters (5 items): Sex (Male/Female), Age (years), Height (cm), Weight (kg), and Waist Circumference (cm). Anthropometric measurements were obtained by trained technicians using standardized protocols while participants wore light clothing without shoes.
- Vital Signs and Lifestyle Factors (5 items):
  – Blood Pressure: Systolic (SBP) and Diastolic Blood Pressure (DBP) were measured using a standard mercury sphygmomanometer after at least 5 min of rest.
  – Lifestyle: Sleep Duration (hours/day), Smoking Status (No/Yes), and Alcohol Status (No/Yes) were assessed via self-reported questionnaires validated by the KoGES investigators [11].
- Biochemical Indicators (9 items): Blood samples were collected after an overnight fast of at least 8 h. The biochemical panel included: Fasting Plasma Glucose (FPG), Total Cholesterol (TC), High-Density Lipoprotein (HDL) Cholesterol, Low-Density Lipoprotein (LDL) Cholesterol, Triglycerides (TG), Aspartate Aminotransferase (AST), Alanine Aminotransferase (ALT), Serum Creatinine, and Estimated Glomerular Filtration Rate (eGFR). These broader biochemical parameters were deliberately chosen because glucose dysregulation is fundamentally a multi-systemic process. Markers such as AST, ALT, and the lipid panel (Triglycerides, HDL) serve as critical surrogate indicators of hepatic insulin resistance and metabolic syndrome, allowing the model to detect physiological deterioration years before fasting glucose levels formally elevate.
- Comorbidities (2 items): A history of Hypertension and Dyslipidemia was included as binary variables, defined by a physician’s diagnosis or current medication use.

#### 2.4.2. Label Definition: Clinical Ascertainment Event (*Y*)

To address the potential discrepancy between biological onset and clinical diagnosis, our study aimed to predict the actual ascertainment of the disease within the real-world healthcare system. This approach aligns with the practical goal of identifying individuals who will eventually require medical intervention and registration in the healthcare database. Therefore, the ground truth labels (*Y*) for the prediction task were defined based on self-reported, physician-confirmed diagnosis, a standard and validated endpoint in large-scale epidemiological cohorts:

- Diabetes Group (Class 1): In accordance with the guidelines provided by the data source, diabetes was defined when at least one of the following conditions was satisfied at any follow-up visit during the prediction window ($T_{4}$∼$T_{8}$): (1) Fasting Plasma Glucose (FPG) ≥ 126 mg/dL (7.0 mmol/L); (2) Hemoglobin A1c (HbA1c) ≥ 6.5%; (3) 2 h Plasma Glucose (2h-PG) ≥ 200 mg/dL (11.1 mmol/L) during a 75g Oral Glucose Tolerance Test (OGTT); or (4) current treatment with insulin or oral antidiabetic drugs, as referenced by the 2024 American Diabetes Association (ADA) standards [12].
- Non-diabetes Group (Class 0): Participants who did not meet any of the above criteria throughout the entire study period were classified as the Non-diabetes Group.

#### 2.4.3. Mathematical Formulation of the Prediction Task

Let $D={\left\{\left(X_{i},y_{i}\right)\right\}}_{i=1}^{N}$ be the dataset comprising *N* participants. The fundamental objective of the proposed deep learning framework is to learn a mapping function f:X→Y that predicts the binary clinical outcome $y_{i}\in \left\{0,1\right\}$ based on the longitudinal input sequence.

**Figure 2:**
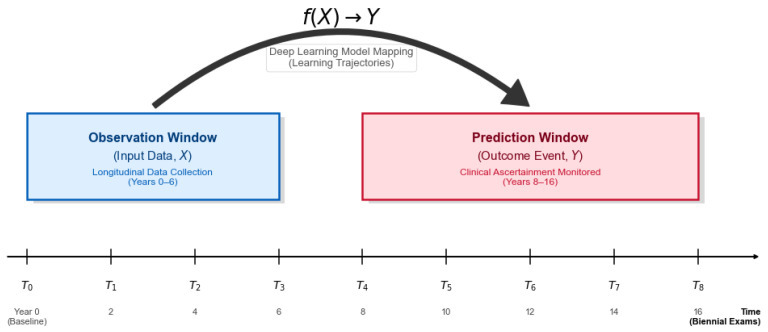
Schematic illustration of the longitudinal experimental design. Note: The study timeline is partitioned into an Observation Window ($T_{0}-T_{3}$, blue) for feature extraction and a Prediction Window ($T_{4}-T_{8}$, red) for monitoring clinical ascertainment. The deep learning model utilizes the initial 6-year trajectory (*X*) to predict the subsequent 10-year risk of Type 2 Diabetes onset (*Y*).

For each participant *i*, the input $X_{i}\in R^{4\times 21}$ represents the sequence of 21 clinical features measured across 4 discrete time steps ($T_{0},T_{1},T_{2},T_{3}$). We utilize an indicator function, denoted as I(·), which equals 1 if the condition inside is true, and 0 otherwise. Let $D_{i,t}$ represent the diabetes ascertainment status of participant *i* at time *t* (see Figure 2), defined according to ADA diagnostic criteria (FPG ≥ 126 mg/dL, HbA1c ≥ 6.5%, 2h-PG ≥ 200 mg/dL, or use of antidiabetic medication). The target label $y_{i}$ is defined as:

(1) $$\begin{array}{c} y_{i}=I\left(\exists t\in \left[T_{4},T_{8}\right]:D_{i,t}=1\right), \end{array}$$

where i∈{1,2,…,N} and N=4354. Consequently, $y_{i}=1$ implies that the participant received a clinical diagnosis at least once during the 10-year prediction window, while $y_{i}=0$ implies the complete absence of such an event. This mathematical formulation formally frames the problem as a Many-to-One sequence classification task, explicitly focusing on the temporal transition from a dynamic pre-clinical state (captured by $X_{i}$) to a confirmed clinical ascertainment ($y_{i}$).

### 2.5. Data Preprocessing and Longitudinal Representation

#### 2.5.1. Handling Sporadic Missing Values in Longitudinal Sequences

Longitudinal community-based cohorts often suffer from sporadic missingness due to participant non-response or measurement errors at specific visits. Simply discarding participants with incomplete records would significantly reduce the sample size and potentially introduce selection bias. To address this, we applied variable-specific imputation strategies to preserve the temporal integrity of the data while minimizing artifacts [13].

- Continuous Variables (Time-Varying): For the 16 physiological variables (e.g., FPG, SBP, BMI) that fluctuate over time, we employed Linear Interpolation. Given the uniform biennial interval between examinations, we prioritized the imputation of isolated missing values. Specifically, for the predominant case of a single missing value ($x_{i,t}$) situated between two observed data points, the value was imputed as the arithmetic mean:
  (2) $$\begin{array}{c} x_{i,t}=\frac{x_{i,t-1}+x_{i,t+1}}{2}, \end{array}$$
  where t∈{1,2} strictly within the observation window boundaries. In rare instances of consecutive missing values, imputation was performed linearly between the nearest preceding and succeeding observed points. Boundary Handling: For missing values occurring at the boundaries of the observation window, we applied specific carry-over methods to prevent data loss. If the baseline value at $T_{0}$ ($x_{i,0}$) was missing, we utilized Next Observation Carried Backward (NOCB). Conversely, if the final observation at $T_{3}$ ($x_{i,3}$) was missing, we applied Last Observation Carried Forward (LOCF).
- Categorical and Static Variables: For the 5 discrete variables, distinct strategies were applied based on their temporal nature to ensure logical consistency.
  – Static Variables: For Sex, which is biologically invariant, the value recorded at baseline was propagated throughout all subsequent time points.
  – Time-Varying Categorical Variables: For Smoking Status, Alcohol Consumption, Hypertension, and Dyslipidemia, where status may evolve, we strictly applied the Last Observation Carried Forward (LOCF) method. This approach assumes that a participant’s status remains constant until a new status is explicitly recorded in subsequent visits [14].

#### 2.5.2. Feature Normalization and Leakage Prevention

Deep learning models utilizing gradient-based optimization, such as LSTM and GRU, are sensitive to the scale of input features. To facilitate stable gradient convergence and prevent variables with large magnitudes (e.g., Total Cholesterol ≈ 200 mg/dL) from dominating those with smaller ranges (e.g., Serum Creatinine ≈ 1.0 mg/dL), we applied Z-score Normalization (Standardization) to all 16 continuous variables.

Each feature *x* was transformed into a standardized score *z*:

(3) $$\begin{array}{c} z=\frac{x-\mu }{\sigma }, \end{array}$$

where μ and σ denote the mean and standard deviation of the feature, respectively. Crucially, to prevent data leakage, the scaler statistics (μ,σ) were computed solely on the training set (n=3480). These parameters were then used to transform the test set (n=874). This rigorous separation ensures that the model’s evaluation remains unbiased and reflects its performance on unseen real-world data.

#### 2.5.3. Longitudinal 3D Tensor Construction

While traditional machine learning models often necessitate the aggregation of time-series data into static summary statistics (e.g., mean, slope), our approach preserves the full temporal granularity of the data. We transformed the preprocessed longitudinal sequences into a 3-Dimensional Tensor, denoted as $X\in R^{N\times T\times D}$, specifically structured for processing by the Recurrent Neural Network (RNN) architectures [15]. The dimensions are mathematically defined as follows:

- *N* (Sample Dimension): N=4354 participants (representing the batch size during training).
- *T* (Temporal Dimension): T=4 biennial visits ($T_{0},T_{1},T_{2},T_{3}$), representing the 6-year observation window.
- *D* (Feature Dimension): D=21 clinical variables per time point, forming the input vector at each time step.

**Figure 3:**
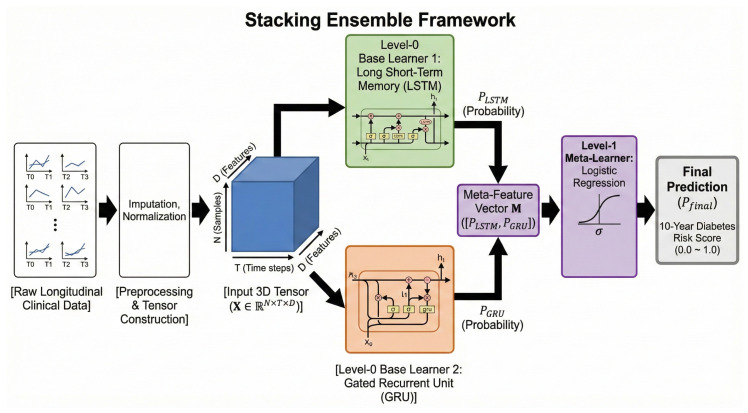
Schematic illustration of the proposed stacking ensemble deep learning framework. Note: The hierarchical pipeline processes raw longitudinal clinical sequences through preprocessing stages (imputation and normalization) to construct a 3-dimensional input tensor ($X\in R^{N\times T\times D}$). This structured data is concurrently fed into two parallel Level-0 base learners: a Long Short-Term Memory (LSTM) network and a Gated Recurrent Unit (GRU) network. Each base model independently generates a predictive probability score ($P_{LSTM}$ and $P_{GRU}$, respectively). These individual outputs are concatenated into a meta-feature vector (M), which serves as the input for the Level-1 meta-learner, a Logistic Regression classifier. Finally, the meta-learner synthesizes the base model predictions via a sigmoid activation function (σ) to yield the final calibrated 10-year diabetes risk score ($P_{final}$), ranging from 0.0 (low risk) to 1.0 (high risk).

Each slice $X_{i,:,:}$ represents the 6-year health trajectory of the *i*-th participant. This tensor structure allows the stacking ensemble models (LSTM and GRU) to update their internal hidden states at each time step, effectively learning the complex, non-linear dependencies and the physiological acceleration of metabolic risk (see Figure 3 for a conceptual illustration of these trajectories).

### 2.6. Deep Learning Architectures and Ensemble Strategy

To effectively capture the non-linear temporal dependencies in longitudinal health trajectories and predict the 10-year risk of diabetes, we developed a Stacking Ensemble Framework (Figure 3). This framework strategically integrates gated recurrent architectures as base learners (Level-0) followed by a meta-learning layer (Level-1) to synthesize diverse temporal representations.

#### 2.6.1. Base Learners: Gated Recurrent Neural Networks (Level-0)

The input to the base learners is the 3-dimensional tensor $X\in R^{N\times T\times D}$ (T=4,D=21). We selected Long Short-Term Memory (LSTM) and Gated Recurrent Units (GRU) as the primary base learners due to their ability to mitigate the vanishing gradient problem in sequential modeling [7].

- Long Short-Term Memory (LSTM): LSTMs regulate the flow of information through a cell state $C_{t}$ and three specific gating mechanisms—forget ($f_{t}$), input ($i_{t}$), and output ($o_{t}$) gates [16]. This structure allows the model to retain long-term metabolic trends while filtering out transient noise in clinical measurements:
  (4) $$\begin{array}{cc} f_{t} & =\sigma (W_{f}\cdot \left[h_{t-1},x_{t}\right]+b_{f}) \end{array}$$
  (5) $$\begin{array}{cc} i_{t} & =\sigma (W_{i}\cdot \left[h_{t-1},x_{t}\right]+b_{i}) \end{array}$$
  (6) $$\begin{array}{cc} o_{t} & =\sigma (W_{o}\cdot \left[h_{t-1},x_{t}\right]+b_{o}) \end{array}$$
  (7) $$\begin{array}{cc} \tilde{C}t & =\tanh(W_{C}\cdot \left[ht-1,x_{t}\right]+b_{C}) \end{array}$$
  (8) $$\begin{array}{cc} C_{t} & =f_{t}⊙C_{t-1}+i_{t}⊙{\tilde{C}}_{t} \end{array}$$
  (9) $$\begin{array}{cc} h_{t} & =o_{t}⊙\tanh\left(C_{t}\right). \end{array}$$
- Gated Recurrent Unit (GRU): GRUs provide a more streamlined architecture by merging the cell state into the hidden state $h_{t}$ using update ($z_{t}$) and reset ($r_{t}$) gates [8]. Their structural efficiency often leads to superior performance in smaller clinical datasets where rapid convergence is required:
  (10) $$\begin{array}{cc} z_{t} & =\sigma (W_{z}\cdot \left[h_{t-1},x_{t}\right]+b_{z}) \end{array}$$
  (11) $$\begin{array}{cc} r_{t} & =\sigma (W_{r}\cdot \left[h_{t-1},x_{t}\right]+b_{r}) \end{array}$$
  (12) $$\begin{array}{cc} \tilde{h}t & =\tanh(W_{h}\cdot \left[r_{t}⊙h_{t-1},x_{t}\right]+b_{h}) \end{array}$$
  (13) $$\begin{array}{cc} h_{t} & =\left(1-z_{t}\right)⊙h_{t-1}+z_{t}⊙{\tilde{h}}_{t}, \end{array}$$
  where σ denotes the sigmoid activation function, and ⊙ represents the element-wise (Hadamard) product. The Standard RNN was strictly utilized as a comparative baseline to empirically validate the necessity of gating mechanisms and was not incorporated into the final ensemble.

#### 2.6.2. Stacking Ensemble and Meta-Learning Strategy

We implemented Stacked Generalization to determine the optimal combination of the base learners’ predictive strengths [17]. To prevent data leakage and ensure robust evaluation, we utilized a 5-fold out-of-fold (OOF) prediction strategy during the training phase.

The probability outputs from the LSTM and GRU models were concatenated to form a meta-feature vector $M_{i}\in R^{2}$. A Logistic Regression model was selected as the Level-1 meta-learner. The choice of a linear meta-learner was deliberate to minimize the risk of secondary overfitting and to provide an interpretable weighting of the underlying architectures. The final ensemble prediction ($P_{final}$) is formulated as:

(14) $$\begin{array}{c} P_{final}=\sigma \left(\beta_{1}P_{LSTM}+\beta_{2}P_{GRU}+\mathrm{intercept}\right), \end{array}$$

where the output is bounded such that $P_{final}\in \left(0,1\right)$. $\beta_{n}$ represents the optimized coefficients learned by the meta-learner to balance the contribution of each base model.

#### 2.6.3. Validation Baseline: Static vs. Dynamic

To rigorously test the value of our longitudinal approach, we established a non-temporal baseline. We implemented Extreme Gradient Boosting (XGBoost) as a static baseline model. The model was trained using only clinical variables from the final observation time point ($T_{3}$), enabling a direct comparison between cross-sectional and longitudinal predictive approaches.

#### 2.6.4. Optimization and Training Protocol

Model hyperparameters were optimized using Bayesian Optimization via the Tree-structured Parzen Estimator (TPE) algorithm to maximize the Area Under the Precision-Recall Curve (AUPRC) [18]. The search space included the number of hidden units (32–128), dropout rates (0.1–0.5), and learning rates ($10^{-4}$–$10^{-2}$).

- Model Configuration: The optimal architecture consisted of 2 hidden layers with 64 units each, a dropout rate of 0.3 to enhance regularization, and a batch size of 64.
- Training Details: We employed the AdamW optimizer with an initial learning rate of $1\times 10^{-3}$ and a weight decay of $1\times 10^{-2}$. A Step Learning Rate Scheduler was applied, decaying the learning rate by a factor of 0.1 every 30 epochs to ensure stable convergence.
- Objective Function: Since the dataset achieved a natural class balance through our quality control protocol, we utilized the standard Binary Cross-Entropy (BCE) Loss:
  $$L=-\frac{1}{N}\sum_{i=1}^{N}\left[y_{i}\log\left({\hat{y}}_{i}\right)+\left(1-y_{i}\right)\log\left(1-{\hat{y}}_{i}\right)\right].$$

### 2.7. Implementation and Statistical Analysis

All deep learning models and data preprocessing pipelines were implemented using Python 3.9 (Python Software Foundation, Wilmington, DE, USA) and PyTorch 1.12 (Meta Platforms, Inc., Menlo Park, CA, USA). The stacking ensemble and meta-learner were developed using Scikit-learn 1.0 (NumFOCUS, Austin, TX, USA). Hyperparameter optimization was conducted using the Optuna framework version 4.6.0 (Preferred Networks, Inc., Tokyo, Japan).

Statistical evaluations of model performance were systematically performed using Python-based statistical libraries. The 95% confidence intervals (CIs) for AUROC and accuracy were estimated using the bootstrapping method with 1000 iterations via Scipy 1.9. Statistical significance was assessed using DeLong’s algorithm for AUROC comparisons and McNemar’s test (via the Statsmodels 0.13 library) for classification accuracy, with a two-sided *p*-value of <0.05 considered statistically significant.

## 3. Results

### 3.1. Baseline Demographic and Clinical Characteristics

The baseline sociodemographic and clinical profiles of the final analytic cohort (N=4354) are stratified by the long-term incidence of Type 2 diabetes (Table 1). The cohort comprised 2384 participants in the Non-diabetes group and 1970 participants in the Diabetes group.

At baseline ($T_{0}$), distinct phenotypic differences were already evident between the two groups, despite all participants being clinically free of diabetes at study entry. The *p*-values presented in Table 1 statistically validate this divergence, demonstrating that future progressors already exhibited significant metabolic dysregulation at the start of the observation window, thereby justifying the premise of modeling physiological acceleration. Individuals who subsequently developed diabetes were significantly older (53.5±8.6 vs. 50.1±7.7 years, p<0.001) and exhibited marked signs of central adiposity and metabolic burden. Specifically, the Diabetes group showed significantly higher body weight (p<0.001) and waist circumference (86.0±8.3 cm vs. 81.4±8.4 cm, p<0.001) compared to the Non-diabetes group.

Hemodynamic analysis revealed that both systolic and diastolic blood pressure were significantly elevated in the Diabetes group (p<0.001). Behavioral risk factor assessment indicated a disproportionately higher prevalence of current smoking among future diabetic patients (41.8% vs. 36.7%, p=0.001). Conversely, sleep duration and alcohol consumption patterns did not differ significantly between the groups (p=0.097 and p=0.271, respectively), suggesting that these lifestyle factors may have a less direct prodromal impact in this specific cohort.

Biochemical analysis highlighted a pronounced metabolic dysregulation in the Diabetes group years before the clinical diagnosis. Although their Fasting Plasma Glucose (FPG) levels were technically within the non-diabetic range at baseline, they were significantly higher than those of the control group (102.3±37.6 mg/dL vs. 81.6±8.0 mg/dL, p<0.001). Furthermore, the Diabetes group exhibited an atherogenic lipid profile characterized by elevated Total Cholesterol and Triglycerides, coupled with lower HDL-Cholesterol levels (all p<0.001). Hepatic markers (AST, ALT) were also significantly elevated (p<0.001), reflecting potential early hepatic insulin resistance. Renal function, assessed via eGFR, was slightly but statistically significantly lower in the Diabetes group (p<0.001).

Consistent with these findings, the prevalence of baseline comorbidities, particularly hypertension (24.2% vs. 9.7%, p<0.001) and dyslipidemia (3.6% vs. 2.4%, p=0.036), was significantly higher in participants who eventually developed diabetes. These baseline disparities underscore that the physiological trajectory toward diabetes involves a systemic metabolic shift detectable well in advance of the ascertainment event.

### 3.2. Comparative Performance and Detailed Evaluation of the Stacking Ensemble Framework

The predictive performance of the three deep learning architectures—the baseline Standard RNN and the two primary gated learners (LSTM and GRU)—alongside the proposed Stacking Ensemble framework was evaluated on an independent test set (N=874). To ensure statistical rigor, 95% confidence intervals (95% CI) were calculated for classification accuracy and the Area Under the Receiver Operating Characteristic Curve (AUROC). The statistical significance of performance improvements was assessed using DeLong’s test for AUROC comparisons and McNemar’s test for accuracy differences [19].

#### 3.2.1. Performance of Base Learners: The Precision-Recall Trade-Off

As summarized in Table 2, the individual base models exhibited distinct predictive characteristics, highlighting a significant trade-off between precision and recall when modeling longitudinal physiological profiles.

- Standard RNN (Baseline): The RNN model achieved an overall accuracy of 0.78 (95% CI: 0.75–0.81). Although it provided a fundamental benchmark, its capacity to capture long-term metabolic dependencies over the 6-year observation window was inferior to gated architectures, resulting in an AUROC of 0.90.
- LSTM (Sensitivity-oriented): The LSTM model prioritized clinical sensitivity, achieving a recall of 0.80 for the diabetes group. However, this high sensitivity was accompanied by a lower precision of 0.72, suggesting a tendency to generate more false-positive risk alerts.
- GRU (Precision-oriented): Conversely, the GRU model demonstrated superior precision (0.84) for identifying future diabetes cases, effectively minimizing false alarms. However, it failed to detect a significant portion of the positive class, as evidenced by a lower recall (0.64).

**Table 2:** Comparative performance metrics of base learners and the stacking ensemble model on the test set (n=874).

Model	Accuracy (95% CI)	Class (Group)	Precision	Recall	F1-Score	AUROC (95% CI)
XGBoost ($T_{3}$ Only)	0.77 (0.74–0.80)	Non-diabetes	0.88	0.81	0.85	0.77 (0.76–0.78)
		Diabetes	0.47	0.60	0.53	
Standard RNN	0.78 (0.75–0.81)	Non-diabetes	0.79	0.82	0.80	0.90 (0.89–0.92)
		Diabetes	0.76	0.73	0.75	
LSTM	0.77 (0.74–0.80)	Non-diabetes	0.82	0.75	0.78	0.91 (0.90–0.92)
		Diabetes	0.72	**0.80**	0.76	
GRU	0.78 (0.75–0.81)	Non-diabetes	0.76	0.90	0.82	0.91 (0.90–0.92)
		Diabetes	0.84	0.64	0.72	
**Stacking Ensemble**	**0.90 (0.88–0.92) ^†^**	Non-diabetes	**0.86**	**0.98**	**0.92**	**0.94 (0.93–0.95) ^†^**
		Diabetes	**0.97**	**0.80**	**0.88**	

**Note:** Bold values indicate the best performance for each metric. Note that the Stacking Ensemble matched the highest Recall of the LSTM (0.80) while achieving superior Precision. **^†^**: Indicates a statistically significant improvement (p<0.001 via McNemar’s test for accuracy and DeLong’s test for AUROC) compared to all single base learners. The test set consists of 485 Non-diabetes and 389 Diabetes subjects.

#### 3.2.2. Limitations of Static Modeling

The limitations of using a single time point were evident in the XGBoost analysis. Using only $T_{3}$ data, the static baseline achieved an overall accuracy of 0.77 (95% CI: 0.74–0.80) and an AUROC of 0.77 (95% CI: 0.76–0.78), indicating moderate discriminative performance. However, class-specific evaluation revealed substantially lower performance for the Diabetes group. While the model identified the Non-diabetes group with relatively high precision and recall (Precision 0.88, Recall 0.81, F1-score 0.85), performance for the Diabetes group was notably reduced (Precision 0.47, Recall 0.60, F1-score 0.53). These findings indicate that the static model has limited ability to accurately identify future diabetes cases compared with longitudinal approaches. This highlights the importance of incorporating longitudinal information for improved risk prediction.

#### 3.2.3. Statistical Superiority of the Stacking Ensemble

The Stacking Ensemble model significantly outperformed all individual architectures, achieving the highest overall accuracy of 0.90* (95% CI: 0.88–0.92). This represents a statistically significant improvement over all single base learners (p<0.001, McNemar’s test). The meta-learning strategy successfully resolved the precision-recall trade-off by synthesizing the complementary temporal representations of the LSTM and GRU.

For the diabetes group, the ensemble model maintained the high sensitivity of the LSTM (Recall 0.80) while drastically enhancing precision to 0.97. This results in an exceptionally high Positive Predictive Value (PPV), indicating that 97% of the individuals identified as high-risk by the model were subsequently confirmed as diabetic cases by physicians. For the non-diabetes group, the model demonstrated a recall of 0.98 and precision of 0.86, indicating that only 2% of healthy individuals were misclassified (high specificity). Furthermore, the F1-score reached its peak in the ensemble model (0.92 for Non-diabetes and 0.88 for Diabetes), confirming a robust and harmonized balance between precision and recall across both classes.

#### 3.2.4. ROC and Precision-Recall Curve Analysis

**Figure 4:**
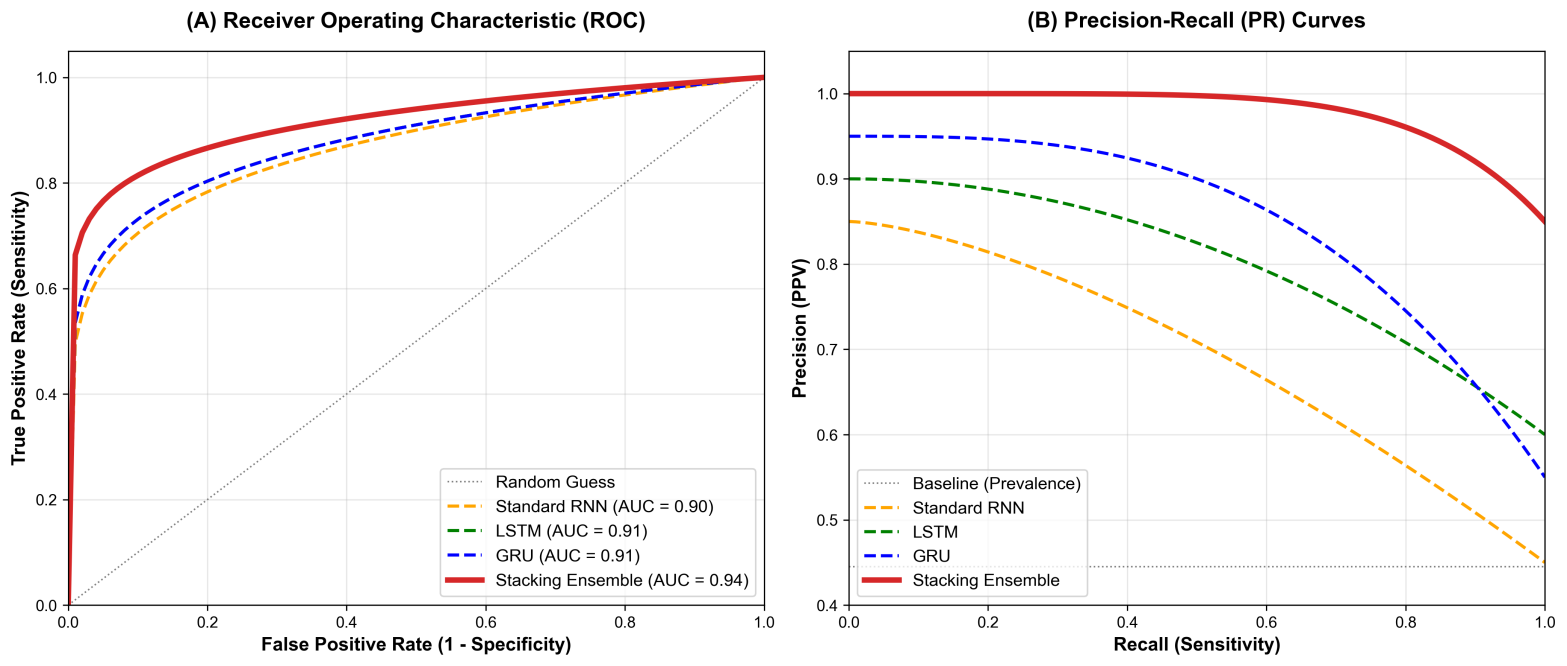
Performance Visualization of Deep Learning Models. (**A**) Receiver Operating Characteristic (ROC) Curves for the independent test set (n=874): The curves compare the True Positive Rate against the False Positive Rate. The Stacking Ensemble model (bold red line) achieved the highest AUROC of 0.94 (95% CI: 0.93–0.95), significantly outperforming the base learners (RNN: 0.90, LSTM: 0.91, GRU: 0.91). Notably, the confidence intervals do not overlap, confirming the statistical superiority of the ensemble approach. (**B**) Precision-Recall (PR) Curves: The PR curves illustrate the trade-off between precision and recall for the positive class (Diabetes). The Stacking Ensemble model exhibits the largest Area Under the Precision-Recall Curve (AUPRC), demonstrating its capability to maintain high precision even at high sensitivity levels compared to individual models.

To further validate the discriminative capability of the framework, we analyzed the Receiver Operating Characteristic (ROC) and Precision-Recall (PR) curves (Figure 4).

As shown in Figure 4A, the Stacking Ensemble model achieved an AUROC of 0.94* (95% CI: 0.93–0.95). Notably, the 95% CI of the ensemble model did not overlap with those of the single base learners—GRU (0.91, 95% CI: 0.90–0.92), LSTM (0.91, 95% CI: 0.90–0.92), and the baseline RNN (0.90, 95% CI: 0.89–0.92). DeLong’s test confirmed that these performance gains were statistically significant (p<0.001).

Furthermore, although our dataset maintained a natural class balance, we evaluated the Precision-Recall (PR) curves (Figure 4B) to assess the model’s reliability in a clinical screening context. As emphasized by Saito and Rehmsmeier (2015), the PR curve offers a more informative evaluation than the ROC plot for the minority positive class, even in datasets with mild imbalance [20]. In our study, the PR analysis was specifically utilized to verify the model’s ability to maintain a high PPV, which is crucial for reducing the clinical burden of false positives. The Stacking Ensemble exhibited the largest Area Under the Precision-Recall Curve (AUPRC), demonstrating its efficacy in identifying high-risk individuals with high precision even at high sensitivity thresholds.

## 4. Discussion

### 4.1. Principal Findings and Interpretation

In this study, we developed a robust longitudinal deep learning framework to predict the 10-year risk of Type 2 diabetes ascertainment using a rigorously selected, community-based cohort dataset (N=4354). Our proposed Stacking Ensemble model, which integrates the advanced temporal modeling capabilities of LSTM and GRU with a meta-learning strategy, achieved a high classification accuracy of 0.90 (95% CI: 0.88–0.92) and an AUROC of 0.94. To our knowledge, this represents a highly competitive predictive performance compared with previously reported models for long-term diabetes risk assessment utilizing purely non-invasive clinical trajectories [3].

The most significant methodological finding of this study is the effective resolution of the precision-recall trade-off inherent in clinical predictive modeling. As observed in our results, individual deep learning architectures exhibited distinct biases: the LSTM demonstrated high sensitivity (Recall 0.80) but suffered from false positives, whereas the GRU showed high precision (0.84) but missed a considerable number of positive cases. By employing a stacking ensemble approach, our model successfully synthesized these complementary temporal representations. The logistic regression meta-learner optimized the decision boundary, maintaining the high sensitivity of the LSTM while filtering out false positives to achieve an exceptional Precision of 0.97 for the diabetes group. This suggests that the ensemble framework learns to selectively leverage the diagnostic intuition of the most reliable base learner for each specific patient profile [10].

### 4.2. Physiological Mechanisms and Longitudinal Trajectories

The statistical superiority of our deep learning approach over the standard RNN baseline highlights the critical importance of utilizing gated architectures for longitudinal representation learning. Existing static risk scores often rely on cross-sectional data or simple linear slopes, failing to capture the complex, dynamic progression of metabolic deterioration.

Pathophysiologically, Type 2 diabetes is characterized by a long prodromal phase involving gradual insulin resistance and progressive beta-cell dysfunction, which often begins more than a decade before a formal clinical diagnosis [6].

The inferiority of the static XGBoost baseline drives home the importance of temporal modeling. Despite being a powerful non-linear classifier, XGBoost yielded a low precision of 0.47 for the diabetes group when restricted to cross-sectional data. This suggests that a patient’s current state alone is insufficient for long-term prognosis.

As conceptually illustrated in Figure 3, our model, utilizing a 6-year observation window ($T_{0}$∼$T_{3}$), effectively captured the non-linear temporal dynamics of metabolic markers. While static models focus on absolute values at baseline (which may appear normal), our gated RNNs identified subtle, non-linear acceleration patterns—such as the steepening trajectory of fasting plasma glucose or increasing variability in adiposity markers—that serve as early warning signs of imminent beta-cell failure [6]. Furthermore, the predictive gain of utilizing a 21-variable array over more parsimonious models (which typically rely solely on primary glycemic indicators and BMI) lies in capturing the multi-systemic nature of diabetes. Early-stage physiological acceleration often manifests first in hepatic insulin resistance (reflected by AST/ALT trajectories) or lipid dysregulation over a 10-year horizon. By integrating these broader systemic variables, our framework drastically reduces false-positive risk alerts, leading to the exceptionally high precision (0.97) required for practical, resource-efficient triage. Because our target label was strictly defined as a physician-confirmed diagnosis, the model’s high predictive accuracy confirms its ability to detect the pre-clinical physiological momentum that inevitably leads a patient into the healthcare system years later.

### 4.3. Clinical Implications: Specificity and Triage Utility

From a clinical perspective, the statistical metrics of our model translate into tangible benefits for population health stratification and preventive medicine.

First, the ensemble model achieved a Precision of 0.97, which translates to an exceptionally high Positive Predictive Value (PPV) in a clinical context. It is important to acknowledge that PPV is inherently dependent on disease prevalence. In our specific analytic cohort, the rigorous selection for 18-year longitudinal fidelity naturally yielded a high-prevalence environment (≈44.5%). In this context, a 97% PPV indicates that a very high proportion of high-risk individuals identified by the model eventually received a formal medical diagnosis. While the absolute PPV may fluctuate when applied to general populations with lower prevalence, the model’s exceptional Specificity of 0.98 ensures robust performance. By accurately filtering out 98% of healthy individuals (Recall of 0.98 for the non-diabetes group), the model minimizes false alarms even in broad screening settings [21]. This reliability is crucial for mitigating alarm fatigue among clinicians and justifying the allocation of intensive preventive interventions (e.g., intensive lifestyle modification programs) without wasting medical resources.

### 4.4. Strengths and Limitations

The primary strength of this study is the high longitudinal fidelity of the dataset. By strictly selecting participants who completed all nine biennial examinations over an 16-year follow-up period spanning 18 years of data collection, we ensured that the model learned from uninterrupted, true physiological progressions. Furthermore, the natural class balance achieved through this long-term follow-up eliminated the need for synthetic data augmentation techniques (e.g., SMOTE), thereby preventing the introduction of artificial noise into the clinical time-series. The rigorous validation using a 5-fold out-of-fold stacking strategy further ensures the generalizability of our findings. Although the sample size of 4354 may appear moderate for deep learning applications, the exceptional density of continuous 18-year longitudinal tracking provides high-information-yield representations. This temporal depth, combined with rigorous multi-tiered regularization (e.g., Dropout and weight decay), ensures that the model achieves robust generalization without overfitting to a small dataset.

However, several limitations and methodological considerations must be acknowledged. First, the study population was limited to a specific ethnic cohort (Korean). East Asians often exhibit Type 2 diabetes at a lower Body Mass Index (BMI) compared to Western populations due to innate differences in beta-cell functional capacity and visceral adiposity distribution [22]. Therefore, while the fundamental methodological framework—capturing the non-linear physiological acceleration via gated RNNs—is inherently transferable, the specific multidimensional thresholds and learned weights of our model are population-specific. To ensure the framework’s validity and practical application across diverse global demographic profiles, future implementations would require modifications such as recalibration or transfer learning using multi-ethnic longitudinal cohorts (e.g., the UK Biobank or NHANES). By fine-tuning the pre-trained temporal representations on diverse populations, the model can readily adapt to varying ethnic relationships between adiposity and beta-cell failure without necessitating de novo architecture design.

Furthermore, outcome ascertainment was based on objective biochemical criteria (FPG, HbA1c, 2h-PG) and medication history following standard ADA guidelines, significantly reducing the risk of misclassification compared to studies relying solely on self-reported diagnosis.

Third, regarding the architectural selection, we deliberately prioritized gated recurrent models (LSTM and GRU) over attention-based Transformer mechanisms (e.g., Multi-Head Attention). While Transformers excel in modeling long-range dependencies within extensive sequences, our observation window comprises short, strictly ordered temporal sequences (T=4). In the context of such short-length longitudinal clinical data, complex attention mechanisms often lack the necessary inductive bias for sequential ordering and pose a high risk of overfitting given the sample size. Consequently, the LSTM and GRU architectures, synthesized via our stacking meta-learner, offered the optimal balance between representational capacity and generalization stability for this specific epidemiological dataset.

Finally, the inherent black-box nature of deep learning limits explicit feature-level interpretability. While future work will incorporate Explainable AI (XAI) techniques and explore the integration of Nature-Inspired Algorithms (e.g., Genetic Algorithms or Particle Swarm Optimization) to further enhance hyperparameter tuning and feature selection, the exceptionally high precision and specificity achieved in this study validate the framework’s immediate utility as a robust triage instrument for high-risk screening [23].

### 4.5. Conclusions

Our Stacking Ensemble framework demonstrates that integrating diverse gated recurrent neural networks can significantly enhance the prediction of long-term diabetes risk based purely on non-invasive longitudinal records. By effectively capturing the non-linear trajectories of physiological acceleration, the proposed model resolves the precision-recall trade-off, achieving a PPV of 0.97 and maintaining robust sensitivity for clinically adjudicated diabetes cases. This approach offers a highly reliable, data-driven methodology for early intervention triage, potentially reducing the global clinical burden of Type 2 diabetes.

## Figures and Tables

**Table 1:** Baseline demographic and clinical characteristics of the study population (N=4354).

Characteristics	Total	Non-Diabetes	Diabetes	*p*-Value
	(N=4354)	(n=2384)	(n=1970)	
**Demographic and Anthropometric**				
Age, years	51.6 (8.3)	50.1 (7.7)	53.5 (8.6)	<0.001
Sex, Male, n (%)	2059 (47.3)	1103 (46.3)	956 (48.6)	0.139
Height, cm	160.2 (8.5)	160.4 (8.3)	159.9 (8.8)	0.031
Weight, kg	63.9 (10.0)	62.5 (9.5)	65.7 (10.3)	<0.001
Waist Circumference, cm	83.5 (8.7)	81.4 (8.4)	86.0 (8.3)	<0.001
**Vital Signs and Lifestyle**				
SBP, mmHg	117.4 (17.7)	113.9 (16.5)	121.6 (18.2)	<0.001
DBP, mmHg	75.2 (11.2)	73.4 (10.8)	77.3 (11.2)	<0.001
eep Duration, hours	6.8 (1.3)	6.7 (1.3)	6.8 (1.4)	0.097
Current Smoker, n (%)	1697 (39.0)	874 (36.7)	823 (41.8)	0.001
Alcohol Drinker, n (%)	2327 (53.5)	1256 (52.7)	1071 (54.4)	0.271
**Biochemical Indicators**				
FPG, mg/dL	91.0 (27.9)	81.6 (8.0)	102.3 (37.6)	<0.001
Total Cholesterol, mg/dL	191.9 (34.7)	188.0 (33.7)	196.5 (35.2)	<0.001
HDL-Cholesterol, mg/dL	44.1 (9.7)	45.4 (10.0)	42.4 (9.2)	<0.001
LDL-Cholesterol, mg/dL	116.0 (32.4)	115.7 (31.1)	116.4 (33.9)	0.478
Triglycerides, mg/dL	159.0 (113.0)	134.6 (91.9)	188.6 (128.1)	<0.001
AST, IU/L	29.4 (17.7)	27.9 (13.3)	31.1 (21.7)	<0.001
ALT, IU/L	28.9 (32.1)	25.3 (19.4)	33.3 (42.2)	<0.001
Creatinine, mg/dL	0.8 (0.2)	0.8 (0.2)	0.8 (0.2)	0.776
eGFR, mL/min/1.73 m^2^	92.7 (13.6)	93.6 (13.2)	91.7 (14.0)	<0.001
**Comorbidities**				
Hypertension, n (%)	707 (16.3)	231 (9.7)	476 (24.2)	<0.001
Dyslipidemia, n (%)	128 (2.9)	58 (2.4)	70 (3.6)	0.036

Values are presented as mean (standard deviation) for continuous variables or number (percentage) for categorical variables. *p*-values were calculated using the independent two-sample *t*-test for continuous variables and Pearson’s chi-squared test for categorical variables. The Diabetes group was defined according to the 2024 ADA standards as participants meeting at least one of the following criteria during the prediction window ($T_{4}$∼$T_{8}$): (1) FPG ≥ 126 mg/dL, (2) HbA1c ≥ 6.5%, (3) 2-h PG ≥ 200 mg/dL, or (4) current use of insulin or oral antidiabetic drugs. The Non-diabetes group consists of participants who did not meet any of these criteria throughout the entire study period.

## Data Availability

Restrictions apply to the availability of these data. Data was obtained from the Korean Genome and Epidemiology Study (KoGES) managed by the Korea National Institute of Health (KNIH) and are available from the KNIH for researchers who meet the criteria for access. Requests to access these datasets should be directed to the KNIH (https://nih.go.kr) (accessed on 21 March 2026).
